# Adaptive AI-generated feedback in higher education: exploring associations with cognitive flexibility and academic psychological safety among engineering students

**DOI:** 10.3389/fpsyg.2026.1820714

**Published:** 2026-08-31

**Authors:** Amani BinJwair

**Affiliations:** Department of Curriculum and Instruction, College of Education, Prince Sattam bin Abdulaziz University, Al-Kharj, Saudi Arabia

**Keywords:** academic psychological safety, adaptive AI-generated feedback, cognitive flexibility, engineering student, higher education, mediation analysis, PLS-SEM, SDG 4: quality education

## Abstract

**Background:**

Adaptive AI-generated feedback (AIF) is increasingly integrated into higher education; however, its cognitive and psychological implications remain insufficiently understood, particularly in engineering education. Grounded in feedback intervention theory and emotion regulation theory, this study developed and tested an integrative model examining the perceived relationships between AIF, cognitive flexibility (CF), and academic psychological safety (APS), including mediating and moderating patterns.

**Methods:**

A cross-sectional survey was conducted among 471 engineering students. Data were analyzed using partial least squares structural equation modeling. The measurement model demonstrated satisfactory reliability and validity, with factor loadings above 0.70, AVE values exceeding 0.50, composite reliability values ranging from 0.908 to 0.936, Cronbach’s alpha values ranging from 0.887 to 0.921, and HTMT values below 0.85. The structural model showed moderate explanatory power for CF (*R^2^* = 0.443) and APS (*R^2^* = 0.626), with adequate predictive relevance and no multicollinearity concerns.

**Results:**

AIF was significantly associated with CF (*β* = 0.580, *p* < 0.001) and APS (*β* = 0.352, *p* < 0.001). CF was significantly associated with APS and showed a significant indirect association between AIF and APS (indirect effect *β* = 0.257, *p* < 0.001). Significant interaction effects indicated that ESAF was associated with a stronger relationship between AIF and CF, whereas MSAF was associated with a stronger relationship between CF and APS.

**Conclusion:**

Perceived AIF was associated with APS directly and indirectly through CF, while emotional and mindfulness-supportive features were associated with a greater strength in these relationships. Given the study’s cross-sectional design, the findings indicate associations rather than causal effects. This study advances the current understanding of cognitive and affective patterns in AI-supported engineering education and highlights the importance of integrating adaptive, emotional, and reflective features into AI feedback systems.

## 1 Introduction

Universities are increasingly integrating AI to support learning and develop adaptive competencies required in the workforce. While early research on AI in education has largely focused on learning outcomes and technology adoption, recent scholarship has called for a broader examination of its cognitive and psychological implications (Bautina, 2025; Elshanhaby et al., 2026). Within this context, adaptive AI-generated feedback (AIF) has emerged as a promising educational tool due to its ability to deliver personalized, iterative, and real-time feedback that supports reflection and self-regulated learning (Ba et al., 2025). Unlike traditional feedback, AIF is typically non-judgmental, immediate, and individualized, which may reshape how students interpret errors and engage in continuous learning cycles.

Engineering education provides a particularly relevant context for investigating these dynamics, as it requires learners to engage with complex, ill-structured problems that demand adaptive reasoning, iterative design thinking, and cognitive restructuring. Cognitive flexibility (CF), defined as the ability to shift thinking strategies, consider alternative solutions, and adapt to changing task demands, has been identified as a critical competence for innovation and success in STEM disciplines (Begum et al., 2025; Chen and Xiao, 2025; Hohl and Dolcos, 2024; Xiong et al., 2025). Prior research suggests that AI-supported learning may encourage flexible thinking by exposing learners to multiple solution pathways and promoting exploratory reasoning (Na-Songkhla et al., 2024; Pop et al., 2025). CF has also been associated with emotional regulation, reduced learning anxiety, and adaptive engagement in digital environments (Fan and Yang, 2026; Karaoglan Yilmaz et al., 2025), highlighting its dual cognitive-affective relevance.

In parallel, academic psychological safety (APS) has received attention as a key psychosocial condition supporting learning. APS refers to students’ perceived ability to express ideas, ask questions, and make mistakes without fear of negative evaluation or embarrassment (Edmondson, 1999; McClintock et al., 2023; McGuire, 2025; Shepard, 2025). It is associated with deeper engagement, critical thinking, and willingness to take intellectual risks.

In AI-supported learning, feedback perceived as non-judgmental and supportive has been shown to reduce evaluative anxiety and encourage experimentation and reflective learning (Chauncey and McKenna, 2024b; Nagata and Uetake, 2025; Peng and Wan, 2025). In addition, emerging research suggests that emotionally supportive AI feedback (ESAF) and mindfulness-supportive AI feedback (MSAF) are associated with improved engagement, emotional regulation, and reduced cognitive anxiety (Elshanhaby et al., 2026; Hacıalioğlu et al., 2025).

ESAF refers to AI-generated feedback designed to regulate learners’ emotional responses by reducing anxiety, minimizing perceived evaluative threat, and enhancing perceived psychological safety (Chauncey and McKenna, 2024a; Gross, 1998). At the affective level, ESAF is theoretically intended to support learners’ emotional interpretation of feedback experiences. It is theorized to be associated with greater learner engagement through reduced defensive responses and emotional resistance, thereby contributing to a more supportive emotional context for learning (Deng and Chen, 2025). In this sense, ESAF is theoretically grounded in emotion regulation theory (Gross, 1998), which emphasizes the role of feedback environments in modulating emotional reactions to evaluation.

MSAF refers to AI-generated feedback that incorporates principles of mindfulness by fostering attentional control, metacognitive awareness, and reflective engagement during learning (Kiye and Çiçek Habeş, 2024; Zeidan et al., 2010). At the metacognitive level, MSAF is intended to support learners’ ability to sustain attention, monitor cognitive processes, and engage in reflective evaluation of their learning strategies. This dimension is aligned with mindfulness-based learning perspectives that emphasize awareness, self-regulation, and cognitive decentering as part of adaptive learning.

Despite recent advances, the ways that AI-generated feedback is associated with cognitive and psychological outcomes remain insufficiently theorized, particularly in higher education (Bautina, 2025; Elshanhaby et al., 2026). Existing literature has primarily focused on behavioral outcomes such as performance, engagement, or technology acceptance, while paying less attention to the underlying psychological processes associated with these outcomes (Alshammari et al., 2026; Alsaiari et al., 2025). Although CF and APS have been examined independently, their interrelationship in AI-mediated learning remains underexplored. Furthermore, limited attention has been paid to how different dimensions of AI-generated feedback are associated with cognitive and psychological outcomes. Consequently, there is limited understanding of how AI feedback relates to cognitive adaptation and psychological safety through integrated psychological factors (Alsaiari et al., 2025; Medina Merodio et al., 2026; Zhao et al., 2026).

To address the gaps outlined above, this study developed and examined a conditional process model based on feedback intervention theory (Kluger and DeNisi, 1996), emotion regulation theory (Gross, 1998), and mindfulness-based perspectives (Zeidan et al., 2010). First, it conceptualized AI-generated feedback as a multidimensional construct comprising distinct cognitive (AIF), affective (ESAF), and metacognitive (MSAF) dimensions rather than treating feedback as a single homogeneous whole. Second, it examined CF as a statistical mediating pathway in the association between AIF to APS. Third, it investigated the conditional roles of emotionally supportive and mindfulness-supportive feedback as boundary conditions influencing key relationships within the model. By doing so, the study provides a more nuanced theoretical explanation of how AI-generated feedback may be associated with learning experiences in AI-supported education. Self-determination theory served as a complementary motivational lens to provide a broader theoretical context but was not directly operationalized or empirically tested.

Feedback intervention theory posits that feedback is most effective when it directs attention toward task-related processes and supports self-regulation rather than self-focused evaluation (Kluger and DeNisi, 1996). Task-focused, specific, and non-judgmental feedback has been associated with the use of adaptive cognitive strategies and improved task performance processes. AIF has been associated with reflection, problem-solving, and the consideration of alternative strategies in prior literature, all of which are theoretically linked to higher cognitive flexibility (Alsaiari et al., 2025). This association informed the first hypothesis of the study:

AIF is positively associated with CF among engineering students.

Emotion regulation theory explains how individuals manage emotional responses to challenging situations (Gross, 1998). In learning, anxiety and fear of negative evaluation are associated with reduced engagement, whereas supportive environments are associated with more adaptive emotional responses. In this study, AIF and ESAF were conceptually linked to emotional regulation. AIF was conceptually associated with reduced evaluative threat, while ESAF was associated with emotional reassurance and perceived psychological support. This led to the second hypothesis:

*H1*: AIF is positively associated with APS among engineering students.

*H2*: In this model, CF was proposed as potentially mediating the relationship between AIF and APS, as articulated in the third hypothesis:

*H3*: CF mediates the relationship between AIF and APS.

Emotion regulation theory predicts that lower emotional distress will be associated with more constructive use of feedback. While AIF is associated with cognitive processing, ESAF is associated with reduced emotional barriers such as stress and defensiveness. This may be associated with more effective engagement with feedback and, consequently, a stronger association between AIF and CF. Empirical evidence suggests that emotionally supportive feedback is associated with both cognitive and psychological learning outcomes (Kraft et al., 2020), thereby informing the fourth hypothesis:

*H4*: ESAF moderates the relationship between AIF and CF, such that the relationship is stronger at higher levels of ESAF.

**Figure 1:**
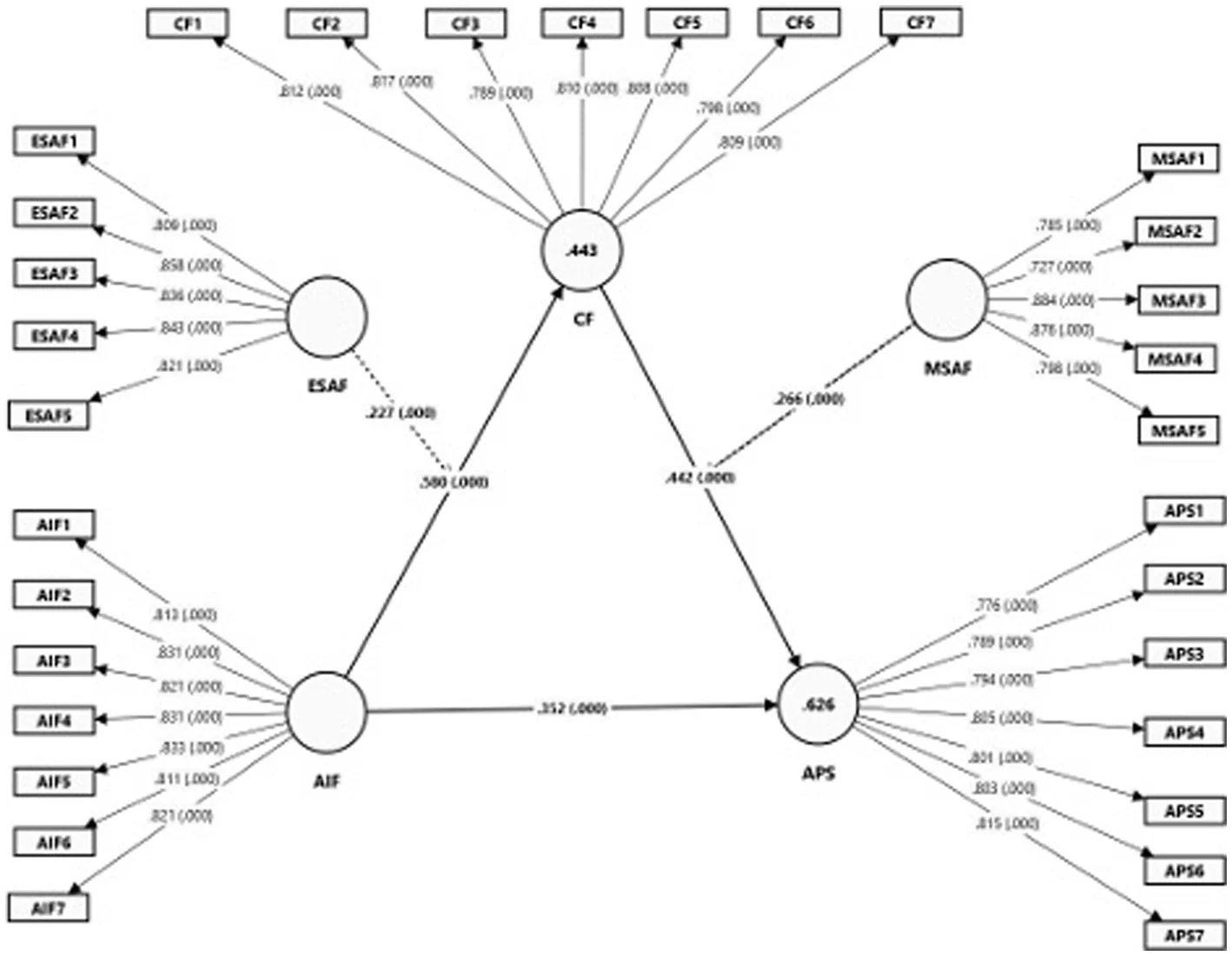
Conceptual model of adaptive AI-generated feedback in engineering education.

MSAF reflects the mindfulness-oriented dimension of AI-generated feedback, emphasizing attention regulation, awareness, and reflective thinking. Mindfulness processes are associated with both cognitive regulation and emotional balance. While CF is associated with adaptive cognitive processing, MSAF is associated with attentional regulation and reflective awareness. These processes may be associated with a stronger positive association between cognitive flexibility and academic psychological safety by supporting attentional regulation and reflective awareness (see Figure 1). This was articulated in the fifth hypothesis:

*H5*: MSAF moderates the relationship between CF and APS, such that the relationship is stronger at higher levels of MSAF.

Under this framework, feedback intervention theory explains the cognitive pathways associated with AIF (Hypotheses 1 and 4), while emotion regulation theory explains the emotional processes associated with APS (Hypotheses 2 and 5).

Consistent with the proposed theoretical framework, the constructs were assigned distinct roles. AIF was specified as the primary cognitive predictor influencing learning outcomes, while ESAF and MSAF were modeled as contextual moderators shaping the strength of relationships between core cognitive factors and outcome variables. ESAF was theorized to be associated with a stronger association between AIF and CF, whereas MSAF was expected to be associated with a stronger association between CF and APS. These theoretical expectations were grounded in the proposed roles of emotional support, attentional regulation, and reflective processing within AI-supported learning.

## 2 Methodology

### 2.1 Design

This study employed a quantitative, cross-sectional design (Creswell and Creswell, 2018). As such, it was limited to examining statistical associations between variables, causal inferences were not claimed, and all relationships were interpreted as correlational and predictive within a structural modeling framework.

### 2.2 Sample

The target population consisted of undergraduate engineering students enrolled at a public university in Saudi Arabia where AI-supported learning tools had been integrated into selected coursework. Purposive sampling was employed to ensure all participants had prior exposure to AIF in authentic learning contexts.

The sample included students from their first through fourth academic years across civil, mechanical, electrical, and computer engineering disciplines. These programs were selected because AI-supported feedback tools had been incorporated into coursework.

To ensure a shared experiential baseline, ChatGPT was used as a formative feedback tool during a structured four-week instructional period. Across participating courses, students engaged with AI-generated feedback during assignments, quizzes, design tasks, and presentations. The AI system was used exclusively for formative purposes, while instructors retained responsibility for summative evaluation.

To standardize exposure, instructors provided a structured prompting framework encouraging students to request feedback on errors, conceptual gaps, and alternative solution strategies. While task content varied across disciplines, all participants followed the same feedback-seeking protocol, ensuring comparability of AI feedback experiences across the sample.

ChatGPT-generated feedback focused primarily on (i) identifying conceptual and procedural errors, (ii) improving reasoning and clarity, and (iii) suggesting alternative approaches. All participants were required to engage with AI feedback at least once per major assignment, ensuring minimum exposure consistency.

Because objective usage logs (e.g., interaction frequency or duration) were not available, the study relied on perceived engagement with AI-generated feedback rather than system-tracked behavioral data. Self-reported frequency of use and prior AI experience were therefore included as proxy indicators of engagement intensity.

### 2.3 Instrument

Data were collected using a structured questionnaire measuring AIF, CF, APS, ESAF, and MSAF. The instrument was developed through a structured adaptation process that integrated established validated measurement instruments, where available, with theory-informed and empirical adaptations derived from recent literature. Specifically, the CF construct was informed by the Cognitive Flexibility Inventory (Dennis and Vander Wal, 2010), while the APS construct was based on the foundational work of Edmondson (1999). To ensure contextual relevance to AI-supported engineering education, selected items were contextually reworded to reflect interactions with AI-generated feedback while preserving their underlying conceptual meaning. For the AI-specific constructs (AIF, ESAF, and MSAF), no universally established validated scales existed at the time of the study, to the best of the author’s knowledge. Therefore, items representing these constructs were synthesized and contextually adapted from recent empirical studies and their theoretical foundations rather than directly adopted from a single validated instrument. Accordingly, no new theoretical constructs were introduced. Rather, context-specific measurement items were developed by synthesizing and adapting existing theoretical concepts, validated measures (where available), and evidence-based indicators to the context of AI-supported engineering education.

The instrument was translated into Arabic using a forward-backward procedure to ensure semantic equivalence. Discrepancies between the original and back-translated versions were resolved through expert consensus.

Content validity was assessed by four experts in educational psychology and educational technology, yielding a scale-level content validity index of 0.92, indicating excellent content validity. Based on their feedback, minor revisions were implemented. A pilot study with 40 engineering students was conducted to assess clarity and preliminary reliability. All items demonstrated acceptable item–total correlations and were retained.

To reduce common method bias, several procedural remedies were implemented, including anonymity assurance, voluntary participation, item randomization, and psychological separation of constructs within the questionnaire. All constructs were measured on a 5-point Likert scale (1 = strongly disagree to 5 = strongly agree) and treated as reflective latent variables. The final questionnaire items, together with their original sources (where applicable) and the empirical or theoretical references informing contextual adaptations, are provided in Appendix 1.

### 2.4 Data collection

Ethical approval was obtained from the Standing Committee of Bioethics Research at Prince Sattam bin Abdulaziz University (Approval No. SCBR-648/2026) before data collection. Participation was voluntary, and informed consent was obtained from all respondents. The questionnaire was administered online over 5 weeks and contained two sections: (i) demographic information (gender, academic level, discipline, prior AI experience, frequency of AI use) and (ii) items for the study constructs.

### 2.5 Data analysis

Unless otherwise noted, data were analyzed using SPSS (Version 30). Exploratory factor analysis was conducted to examine the underlying factor structure of the adapted measurement items prior to confirmatory assessment. The suitability of the data was confirmed using the Kaiser–Meyer–Olkin measure and Bartlett’s test of sphericity (Field, 2009; Pallant, 2010). Factor extraction was based on eigenvalues greater than 1.0 and cumulative variance exceeding 60% as recommended in the literature (Hair et al., 2010; Field, 2009; Tabachnick and Fidell, 2006; Thompson and Daniel, 1996). Promax oblique rotation was applied due to expected correlations between constructs. A minimum loading threshold of 0.40 was adopted. All items met acceptable criteria, and no items were removed. Although exploratory factor analysis and partial least squares structural equation modeling (PLS-SEM) were conducted on the same dataset, exploratory factor analysis was used as an initial exploratory step because measurement scales were adapted to an AI-supported learning context. However, the absence of an independent validation sample should be considered when interpreting measurement robustness.

PLS-SEM was conducted using SmartPLS 4 to examine the proposed conceptual model, including direct, mediating, and moderating associations. PLS-SEM was selected due to its suitability for complex models with latent constructs and its robustness in exploratory and prediction-oriented research (Hair et al., 2022; Henseler et al., 2015). The analysis followed a two-stage procedure: (i) assessment of the measurement model (reliability and validity) and (ii) evaluation of the structural model (path coefficients, mediation, moderation, and predictive power).

Although global fit indices (e.g., SRMR, NFI) were reported as Supplementary material, model evaluation primarily relied on variance-based criteria (*R*^2^, *f*^2^, *Q*^2^), consistent with best practices in PLS-SEM. Since AIF, ESAF, and MSAF were conceptually modeled as distinct but related dimensions, discriminant validity was assessed both statistically (HTMT, Fornell–Larcker) and theoretically.

## 3 Results

### 3.1 Descriptive statistics

The demographic profile is presented in Table 1. The sample was predominantly male (80.7%) and undergraduate (73.7%). Regarding prior experience with AI tools, 74.9% reported limited experience, and 17.2% reported high experience. Regarding AI-generated feedback use, most reported frequent use: 74.1% “often” and 11.0% “always.” These characteristics indicated that participants reported regular engagement with AI-supported learning, providing an appropriate context for examining the proposed associations.

**Table 1:** Respondent profile.

Characteristic	Category	*N*	%
Gender	Male	380	80.7
	Female	91	19.3
Education	Diploma	89	18.9
	Bachelor’s	347	73.7
	Master’s	35	7.4
Prior AI experience	None	37	7.9
	Limited	353	74.9
	High	81	17.2
Frequency of AI feedback use	Always	52	11.0
	Often	349	74.1
	Sometimes	40	8.5
	Rarely	17	3.6
	Never	13	2.8

### 3.2 Measurement model evaluation

#### 3.2.1 Exploratory factor analysis

The results of the exploratory factor analysis are presented in Tables 2, 3. The Kaiser–Meyer–Olkin measure of sampling adequacy was 0.904, and Bartlett’s test of sphericity was significant (*χ*^2^ = 8317.80, *p* < 0.001), confirming the suitability of the data for factor analysis. As shown in Table 2, five factors with eigenvalues greater than 1.0 were retained according to Kaiser’s criterion, jointly accounting for 88.2% of the total variance.

**Table 2:** Exploratory factor analysis: factor extraction results.

Factor	Eigenvalue	Proportion of variance	Cumulative proportion	Factor retention
Factor 1	4.686	15.12%	15.12%	Retained
Factor 2	4.264	13.76%	28.88%	Retained
Factor 3	3.959	12.77%	41.65%	Retained
Factor 4	3.003	9.68%	51.33%	Retained
Factor 5	2.751	8.87%	60.20%	Retained
Factor 6	0.138	0.44%	60.64%	Not retained
Factor 7	0.112	0.36%	61.00%	Not retained

Factor retention was based on the Kaiser criterion (eigenvalue > 1), supported by cumulative variance exceeding 60% and theoretical interpretability of the extracted factors.

**Table 3:** Rotated factor loadings and communalities for the retained measurement items.

Factor	Item	Rotated loading (AIF)	Communality (h^2^)
Factor 1 (AIF)	AIF1	0.796	0.632
	AIF2	0.779	0.637
	AIF3	0.772	0.655
	AIF4	0.768	0.627
	AIF5	0.793	0.655
	AIF6	0.794	0.641
	AIF7	0.784	0.672
Factor 2 (CF)	CF1	0.214	0.646
	CF2	0.203	0.599
	CF3	0.210	0.611
	CF4	0.220	0.597
	CF5	0.208	0.583
	CF6	0.212	0.612
	CF7	0.215	0.608
Factor 3 (APS)	APS1	0.132	0.626
	APS2	0.140	0.587
	APS3	0.145	0.648
	APS4	0.138	0.557
	APS5	0.142	0.589
	APS6	0.136	0.594
	APS7	0.139	0.642
Factor 4 (ESAF)	ESAF1	0.165	0.598
	ESAF2	0.160	0.635
	ESAF3	0.158	0.627
	ESAF4	0.162	0.628
	ESAF5	0.159	0.609
Factor 5 (MSAF)	MSAF1	0.160	0.612
	MSAF2	0.158	0.622
	MSAF3	0.162	0.578
	MSAF4	0.165	0.638
	MSAF5	0.160	0.634

*h*^2^ = Communality. Only primary factor loadings are reported. Factor retention was based on the Kaiser criterion (eigenvalues > 1), yielding a five-factor structure.

The rotated factor solution reported in Table 3 supported a clear five-factor structure consistent with the proposed measurement model. All items loaded most strongly on their intended constructs, with primary factor loadings ranging from 0.736 to 0.830 and communalities ranging from 0.557 to 0.672. These results provided preliminary evidence of factorial validity and justified retaining all measurement items for subsequent analysis.

#### 3.2.2 Convergent validity, reliability, and discriminant validity

The measurement model results are presented in Tables 4, 5. All factor loadings exceeded the recommended threshold of 0.50, with most exceeding 0.80. The average variance extracted (AVE) values ranged from 0.636 to 0.695, confirming adequate convergent validity. Internal consistency reliability was also established, with Cronbach’s alpha values ranging from 0.887 to 0.921 and composite reliability (CR) values ranging from 0.908 to 0.936, all exceeding the recommended thresholds.

**Table 4:** Convergent validity and reliability assessment of the measurement model.

Latent constructs	Items	Factor loading	*AVE*	*CR*	Cronbach’s alpha
Adaptive AI-generated feedback (AIF)	AIF1	0.813	0.677	0.936	0.921
	AIF2	0.831			
	AIF3	0.821			
	AIF4	0.831			
	AIF5	0.833			
	AIF6	0.811			
	AIF7	0.821			
Cognitive Flexibility (CF)	CF1	0.812	0.650	0.929	0.910
	CF2	0.817			
	CF3	0.789			
	CF4	0.810			
	CF5	0.808			
	CF6	0.798			
	CF7	0.809			
Academic Psychological Safety (APS)	APS1	0.776	0.636	0.924	0.905
	APS2	0.789			
	APS3	0.794			
	APS4	0.805			
	APS5	0.801			
	APS6	0.803			
	APS7	0.815			
Emotionally Supportive AI Feedback (ESAF)	ESAF1	0.809	0.695	0.919	0.892
	ESAF2	0.858			
	ESAF3	0.836			
	ESAF4	0.843			
	ESAF5	0.821			
Mindfulness-Supportive AI Feedback (MSAF)	MSAF1	0.785	0.666	0.908	0.887
	MSAF2	0.727			
	MSAF3	0.884			
	MSAF4	0.876			
	MSAF5	0.798			

AVE, Average variance extracted; CR, Composite reliability. Factor loadings represent the standardized loadings of the measurement items on their respective constructs.

**Table 5:** Discriminant validity assessment (Fornell–Larcker criterion and HTMT ratios).

Latent constructs	Fornell-Larcker criterion				
	AIF	APS	CF	ESAF	MSAF
AIF	0.823				
APS	0.638	0.798			
CF	0.595	0.688	0.806		
ESAF	0.006	0.081	0.194	0.834	
MSAF	−0.032	0.087	0.043	0.235	0.816

Latent constructs	HTMT criterion				
	AIF	APS	CF	ESAF	MSAF
APS	0.698				
CF	0.648	0.757			
ESAF	0.038	0.088	0.207		
MSAF	0.075	0.077	0.052	0.256	

AVE, Average variance extracted; HTMT, Heterotrait–Monotrait ratio of correlations. Diagonal values in the Fornell–Larcker matrix represent the square roots of AVE for each construct.

Discriminant validity was confirmed using both the Fornell–Larcker criterion (Fornell and Larcker, 1981) and the heterotrait–monotrait ratio (HTMT). All HTMT values were below the recommended threshold of 0.85 (Hair et al., 2022), and the square roots of the AVE exceeded the corresponding inter-construct correlations. As shown in Table 5, stronger correlations were observed between theoretically related constructs in the structural model (AIF–APS, AIF–CF, and CF–APS), whereas the correlations among the AI feedback dimensions (AIF, ESAF, and MSAF) were generally low. These results indicated that the latent constructs were empirically distinct and supported the adequacy of the measurement model.

#### 3.2.3 Common method bias

Common method bias was assessed using Harman’s single-factor test and full collinearity VIFs. Results indicated that no single factor accounted for the majority of variance, and all VIF values were below 3.0, suggesting that common method bias was unlikely to be a serious threat. However, since all variables were collected using self-reported data at a single point in time, potential residual bias could not be fully excluded, and results should be interpreted with appropriate caution.

#### 3.2.4 Structural model evaluation

The structural model was evaluated using PLS-SEM. As shown in Table 6, the model demonstrated acceptable model quality indicators. The SRMR value was 0.038 (< 0.08), indicating a low discrepancy between observed and model-implied matrices (Henseler et al., 2014; Hu and Bentler, 1999). In addition, the NFI value was 0.921 (> 0.90), further confirming model adequacy (Bentler and Bonett, 1980; Henseler et al., 2016).

**Table 6:** Model fit indices.

Fit index	Value	Recommended threshold	Interpretation
SRMR (standardized root mean square residual)	0.038	Less than 0.08	Good fit
NFI (Normed Fit Index)	0.921	Greater than 0.90	Acceptable fit

SRMR, Standardized root mean square residual; NFI, Normed fit index.

**Figure 2:**
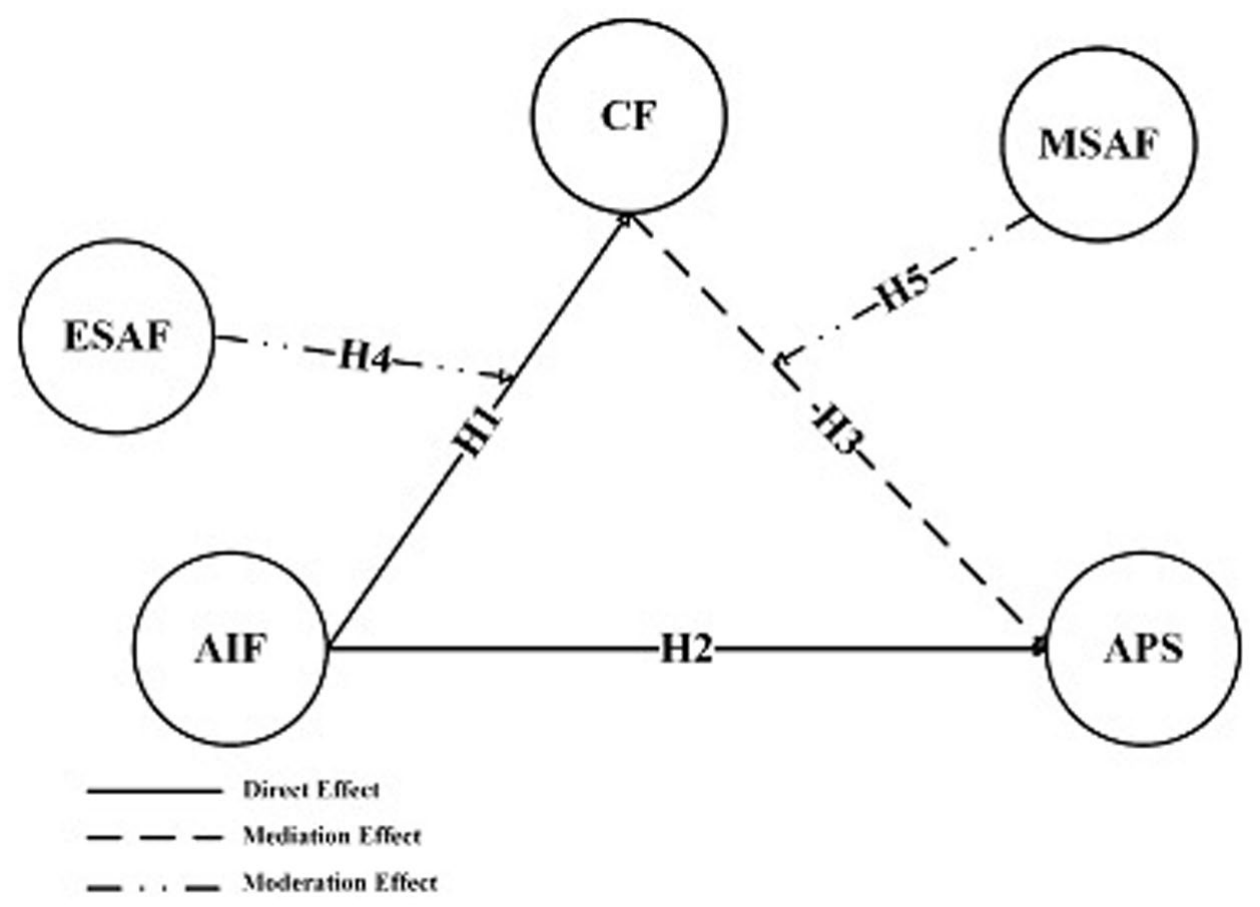
PLS-SEM structural model with standardized path coefficients and factor loadings.

Multicollinearity was not an issue, as all VIF values were below 3.0 (see Table 7). The model explained substantial variance in CF (*R*^2^ = 0.443) and APS (*R*^2^ = 0.626). Predictive relevance was confirmed with *Q*^2^ values of 0.286 (CF) and 0.395 (APS). The full structural model is presented in Figure 2.

**Table 7:** Structural path coefficients and hypothesis testing results.

Path	*β*	*SD*	*t*	*p*	*f* ^2^	*VIF*	Result
AIF → CF	0.580	0.030	19.365	0.000	0.603	1.004	H_1_ supported
AIF → APS	0.352	0.036	9.822	0.000	0.212	1.559	H_2_ supported

β, standardized path coefficient; SD, Standard deviation; *f*^2^, effect size; VIF, Variance inflation factor.

#### 3.2.5 Direct effect size

Bootstrapping results (5,000 resamples) are presented in Table 7 and Figure 2. AIF was significantly associated with CF (*β* = 0.580, *p* < 0.001), supporting Hypothesis 1. The effect size was large (*f^2^* = 0.603). AIF was also significantly associated with APS (*β* = 0.352, *p* < 0.001), supporting Hypothesis 2, with a moderate effect size (*f^2^* = 0.212).

#### 3.2.6 Mediation effect size

As shown in Table 8 and Figure 2, AIF’s indirect association with APS via CF was significant (*β* = 0.257, *p* < 0.001). Since both direct and indirect effect sizes were significant, the mediation was classified as partial complementary, providing statistical support for Hypothesis 3.

**Table 8:** Mediation analysis results.

Indirect effect	*β*	*SD*	*t*	*p*	Mediation type	Result
AIF→ CF→ APS	0.257	0.026	10.057	0.000	Partial, complementary	H_3_ supported

β, Standardized path coefficient; SD, Standard deviation.

#### 3.2.7 Moderation effect size

**Figure 3:**
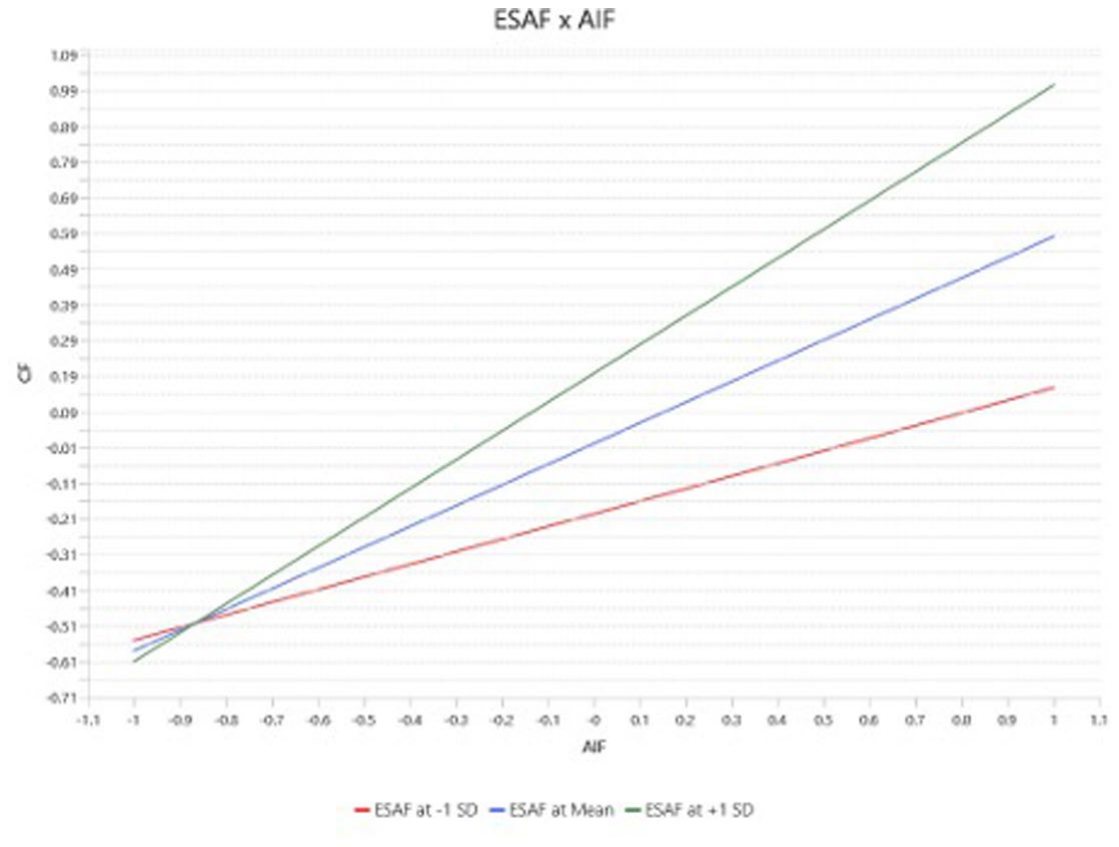
Moderating effect of ESAF on the relationship between AIF and CF.

**Figure 4:**
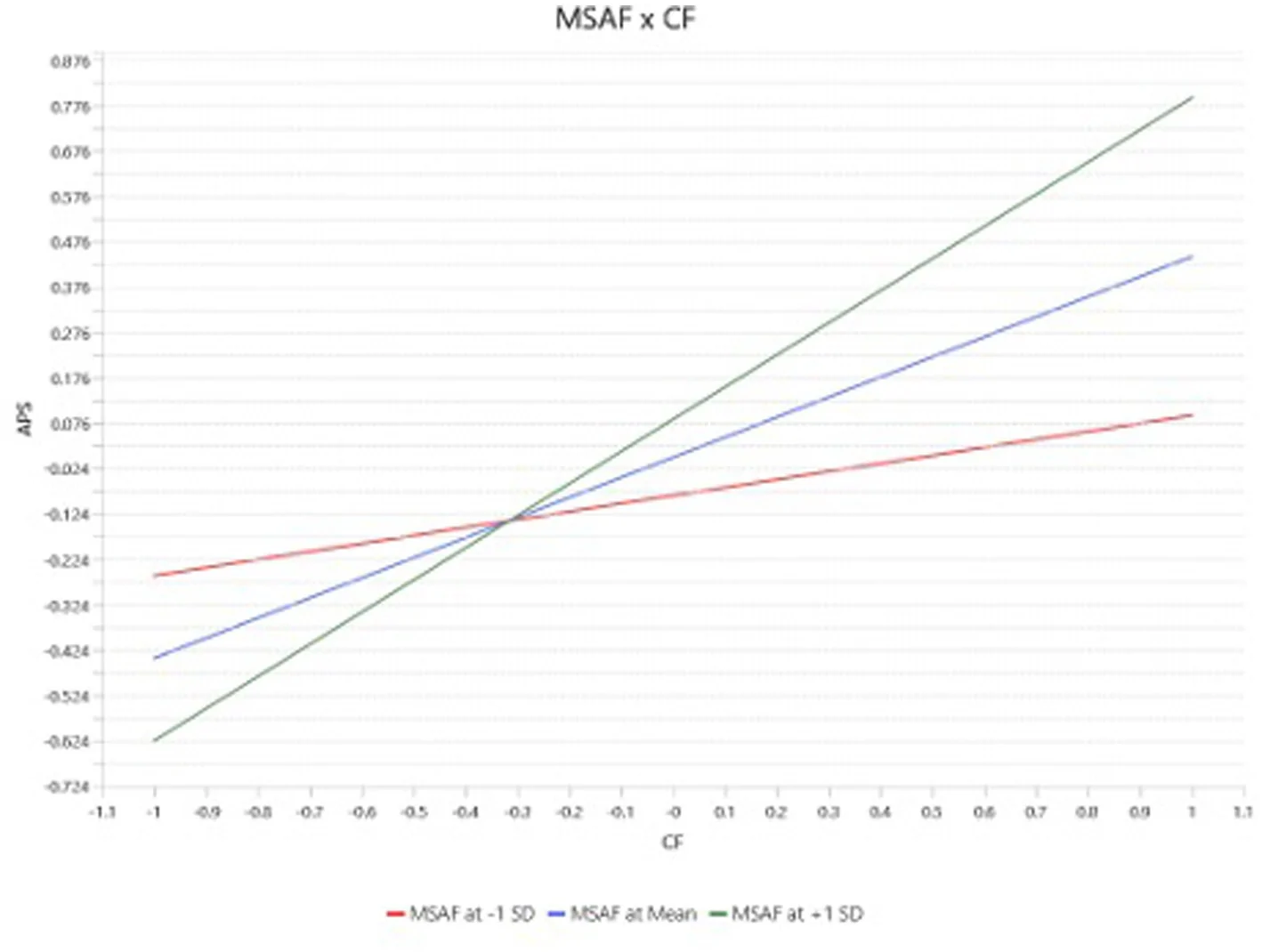
Moderating effect of MSAF on the relationship between CF and APS.

Moderation results are presented in Table 9 and Figures 3, 4. ESAF significantly moderated the relationship between AIF and CF (*β* = 0.227, *p* < 0.001), providing statistical support for Hypothesis 4, with a small effect size (*f*^2^ = 0.095). MSAF significantly moderated the relationship between CF and APS (*β* = 0.266, *p* < 0.001), supporting Hypothesis 5, with a moderate effect size (*f^2^* = 0.175). In addition, ESAF had a small but significant association with CF, while MSAF had a negligible association with APS. Overall, both moderating factors were associated with variations in the strength of the proposed relationships within the model.

**Table 9:** Moderation analysis results.

Path	*β*	SD	*t*	*p*	*f* ^2^	VIF	Result
ESAF → CF	0.197	0.035	5.615	0.000	0.070	1.001	
ESAF x AIF → CF	0.227	0.031	7.404	0.000	0.095	1.004	H_4_ supported
MSAF → APS	0.084	0.040	2.090	0.037	0.019	1.007	
MSAF x CF → APS	0.266	0.058	4.612	0.000	0.175	1.017	H_5_ supported

*β*, Standardized path coefficient; SD, Standard deviation; *f*^2^, effect size; VIF, Variance inflation factor.

## 4 Discussion

The results provided empirical support for the proposed statistical associations (Hypotheses 1–5), with the model demonstrating substantial explanatory power for CF (*R^2^* = 0.443) and APS (*R^2^* = 0.626). These findings indicate significant statistical associations among perceived AI-generated feedback dimensions, cognitive flexibility, and academic psychological safety; however, given the cross-sectional and self-reported design, any relationships should be interpreted as associative rather than causal.

### 4.1 Multidimensional structure of AI-generated feedback

The findings provided empirical support for a multidimensional conceptualization of AI-generated feedback comprising cognitive (AIF), emotional (ESAF), and metacognitive (MSAF) dimensions. Although discriminant validity was confirmed, the results indicated that AIF, ESAF, and MSAF represented empirically distinct dimensions, as demonstrated by the low inter-construct correlations and HTMT values reported in Table 5.

This finding suggested that learners may perceive cognitive, emotional, and metacognitive aspects of AI-generated feedback as differentiated features rather than overlapping components of a single underlying construct. This interpretation was consistent with recent research emphasizing that AI-generated feedback may involve multiple dimensions related to cognitive adaptation, emotional experiences, and reflective learning processes (Alsaiari et al., 2025; Alsaiari et al., 2025; Nagata and Uetake, 2025). Unlike interpretations based on high conceptual overlap, the present findings provide empirical support for maintaining AIF, ESAF, and MSAF as separate first-order constructs. This distinction contributes to ongoing discussions regarding the dimensional structure of AI-mediated feedback by demonstrating that theoretically related dimensions can remain statistically distinguishable. Although a higher-order conceptualization of AI-generated feedback remains theoretically possible, the current findings did not demonstrate substantial empirical overlap among AIF, ESAF, and MSAF. Therefore, a higher-order model was not examined because the primary objective of this study was to investigate the differentiated relationships of each dimension with CF and APS. Future research may compare first-order, second-order, and bifactor models across different educational contexts to determine the most appropriate representation of AI-generated feedback constructs.

### 4.2 AIF and CF (hypothesis 1)

AIF exhibited a strong positive association with CF (*β* = 0.580, *p* < 0.001, *f^2^* = 0.603), representing the strongest statistical association in the model. This finding indicated that students who perceived AI feedback as more adaptive and cognitively supportive also tended to report higher levels of CF. The result was consistent with feedback intervention theory, which proposes that effective feedback can support task-focused cognitive restructuring and self-regulation processes (Kluger and DeNisi, 1996). It also aligned with empirical studies by Chauncey and McKenna (2024a), Na-Songkhla et al. (2024), and Pop et al. (2025), which found that AI-supported learning environments were associated with adaptive thinking, reflective reasoning, and problem reconfiguration. The finding is particularly relevant in engineering education, where complex problem-solving tasks often require learners to consider alternative approaches and revise existing solutions. However, the present cross-sectional evidence does not establish that AIF causes increases in CF; rather, it indicates a significant association between perceived AIF and CF.

### 4.3 AIF and APS (hypothesis 2)

AIF demonstrated a significant positive relationship with APS (*β* = 0.352, *p* < 0.001, *f^2^* = 0.212), with students who reported higher levels of AIF also reporting higher levels of APS. This aligned with prior studies suggesting that AI-mediated feedback may support learner comfort and reduce concerns related to evaluation in learning (e.g., Nagata and Uetake, 2025; Peng and Wan, 2025). Similarly, Chauncey and McKenna (2024a) suggested that AI systems may provide opportunities for less threatening and more accessible feedback interactions. The present study extends this literature by indicating that perceived AIF was statistically associated with APS while recognizing that the direction and temporal nature of this relationship require further investigation.

### 4.4 Mediating role of CF (hypothesis 3)

CF showed a significant partial mediation effect in the association between AIF and APS (*β* = 0.257, *p* < 0.001). This finding suggested that CF represented an explanatory pathway through which perceptions of AIF were associated with APS. This finding extends prior studies that examined the relationships between AI feedback and cognitive or affective outcomes (Alsaiari et al., 2025; Pop et al., 2025). The results indicated that students who perceived AI feedback as cognitively supportive may also perceive greater opportunities to reconsider information, explore alternative approaches, and adapt their thinking, which are processes associated with CF. However, this apparent mediation should not be interpreted as evidence of a causal mechanism because the data were collected at a single point in time. Instead, the findings identify an empirical pathway that warrants further examination through longitudinal or experimental research. The coexistence of significant direct and indirect effect sizes suggested that the relationship between AIF and APS may involve multiple complementary associations, including cognitive processes represented by CF.

### 4.5 Moderating role of ESAF (hypothesis 4)

ESAF significantly moderated the relationship between AIF and CF (*β* = 0.227, *p* < 0.001), albeit with a small effect size (*f^2^* = 0.095). This finding indicated that the strength of the association between AIF perceptions and CF varied according to students’ perceptions of emotional support within AI-generated feedback. This was consistent with Chauncey and McKenna (2024a) and Nagata and Uetake (2025), who emphasized the importance of emotional dimensions in AI-supported learning. Recent research further suggests that emotionally enriched AI feedback may support learner well-being and engagement without necessarily replacing the cognitive aspects of feedback (Alsaiari et al., 2025). The present findings refine this perspective by suggesting that, rather than being a dominant predictor of cognitive outcomes, ESAF was associated with a stronger relationship between AIF and CF, which may indicate that supportive emotional features may be associated with greater engagement with AIF. However, the data did not demonstrate that ESAF reduced emotional barriers or produced changes in CF.

### 4.6 Moderating role of MSAF (hypothesis 5)

MSAF significantly moderated the relationship between CF and APS (*β* = 0.266, *p* < 0.001), with a moderate effect size (*f^2^* = 0.175). This finding suggested that the association between CF and APS differed depending on students’ perceptions of mindfulness-supportive AI feedback. This aligned with mindfulness-based learning theories, which emphasize attentional regulation and reflective awareness as important processes supporting adaptive learning (Kiye and Çiçek Habeş, 2024; Zeidan et al., 2010). The result was also consistent with emerging research suggesting that metacognitive and reflective processes may influence how learners engage with AI-supported learning (Worapongpat, 2025). However, the relatively weak direct effect size suggested that MSAF was primarily associated with a stronger link between CF and APS rather than acting as a standalone predictor. Accordingly, MSAF may be interpreted as a contextual factor associated with the extent to which CF corresponds with APS. Nevertheless, this moderating association represents a statistical interaction rather than evidence that mindfulness-oriented AI feedback directly produces psychological benefits.

### 4.7 Integrative interpretation and theoretical implications

According to the results, AIF represented a task- and cognition-oriented factor, ESAF an affective-regulatory factor, and MSAF a metacognitive-attentional factor. From a structural perspective, the model demonstrated substantial explanatory power, accounting for 44.3% of the variance in CF and 62.6% in APS. This indicated that the framework captured meaningful variability in learners’ cognitive and psychological outcomes in AI-supported learning.

The discriminant validity results further clarify the dimensional structure of AI-generated feedback. Although AIF, ESAF, and MSAF are theoretically related dimensions within AI-supported learning, the low inter-construct correlations and HTMT values reported in Table 5 indicated that they represent empirically distinct constructs rather than highly overlapping components of a single experience.

Consistent with feedback intervention theory (Kluger and DeNisi, 1996), the findings suggested that the cognitive features of AIF within the proposed model were associated with higher reported cognitive flexibility, which involves considering alternative solution pathways and adapting problem-solving strategies. More specifically, the significant association between AIF and CF suggested that learners who perceived AI feedback as adaptive and cognitively supportive also reported higher CF. This interpretation extends previous research by highlighting the relevance of cognitive adaptation processes in AI-supported learning (Alsaiari et al., 2025; Chauncey and McKenna, 2024a; Pop et al., 2025).

Within the proposed model, AIF was associated with CF, and CF statistically mediated the association between AIF and APS. Thus, CF could help explain why learners’ perceptions of AIF were associated with higher levels of APS. The persistence of a significant direct association between AIF and APS indicated that CF did not fully explain the relationship between perceived AIF and APS. However, because the study was cross-sectional, these findings should be interpreted as evidence of statistical associations rather than confirmation of sequential causal processes.

In line with emotion regulation theory, the findings indicated that perceptions of emotional support were associated with differences in learners’ engagement with AIF. Rather than suggesting that ESAF directly reduced anxiety or emotional barriers, the moderation results indicated that emotional support perceptions were associated with a stronger relationship between AIF and CF. This finding supported the view that affective features may provide a supportive context when engaging with AI feedback (Alsaiari et al., 2025; Gross, 1998).

MSAF was associated with a stronger relationship between CF and APS. This moderating relationship suggested that mindfulness-supportive feedback may be associated with differences in how cognitive flexibility relates to perceptions of psychological safety. However, this statistical relationship did not establish that MSAF independently generated psychological outcomes.

Taken together, the findings supported a multidimensional psychological framework in which AI-generated feedback can be examined as involving interconnected yet conceptually distinct cognitive, emotional, and metacognitive dimensions. Future research could empirically compare alternative measurement structures, including higher-order and bifactor models, using independent datasets. This integrated structure extends existing literature by moving beyond single-pathway explanations of AI feedback. The results suggested that AI-generated feedback could be examined as a multidimensional learning support system in which different perceived feedback characteristics show distinct statistical relationships with learners’ cognitive and psychological experiences.

### 4.8 Theoretical contributions

First, this study extends feedback intervention theory by suggesting that AIF was positively associated with CF, which in turn partially mediated its relationship with APS through a significant complementary mediation pathway. Thus, CF was found to be an important explanatory variable linking perceptions of AIF with APS outcomes. However, the results should be understood as relational evidence rather than confirmation of a causal mechanism. These findings position CF as linking feedback perception to psychological outcomes in AI-supported learning, extending prior applications of feedback theory to generative AI (Chauncey and McKenna, 2024a; Kluger and DeNisi, 1996; Pop et al., 2025).

Second, the study extends emotion regulation theory by showing that ESAF strengthened the relationship between AIF and CF. The findings indicated that emotional support represented a moderating association between AIF perceptions and CF. This provides a more nuanced interpretation than treating emotional support as a direct driver of learning outcomes. Accordingly, the study highlights the importance of affective design features in AI-mediated learning (Alsaiari et al., 2025; Gross, 1998; Nagata and Uetake, 2025).

Third, the study contributes to mindfulness-based learning perspectives by showing that MSAF was associated with a stronger relationship between CF and APS, suggesting mindfulness-oriented feedback characteristics may shape this relationship, while their role as an independent predictor appeared limited. This refines existing perspectives on attentional regulation and reflective awareness in technology-enhanced learning (Kiye and Çiçek Habeş, 2024; Zeidan et al., 2010).

Fourth, the study supported ongoing efforts to refine the dimensional structure of AI-feedback perceptions and provides a foundation for future higher-order and rival-model testing. Given the discriminant validity results reported in Table 5, future studies could empirically determine whether AI-feedback dimensions are better represented as separate constructs or components of a broader theoretical framework.

Finally, the study extends AI-feedback research in higher education by shifting the focus from purely performance-oriented outcomes toward an integrated cognitive–affective–metacognitive framework.

### 4.9 Practical implications

The findings offer practical considerations for the design and integration of AI-generated feedback in engineering education. Because the study examined learners’ perceptions rather than objective system characteristics, these implications should be interpreted as design considerations rather than demonstrated effects of specific AI features. AI feedback systems may benefit from prioritizing adaptive and iterative structures that extend beyond correctness evaluation and are aligned with learners’ CF. Design approaches may incorporate prompts that support alternative reasoning, reflection, and solution exploration, as these features are consistent with the observed association between AIF and CF.

In addition, the results highlight the importance of incorporating affective design features into AI feedback systems. However, the low correlations among AIF, ESAF, and MSAF indicated that emotional support should not be considered simply a component overlapping with AIF. Instead, ESAF represents a distinct dimension that may complement cognitive feedback functions. MSAF was associated with a stronger relationship between CF and APS. Therefore, reflective prompts and metacognitive features may be considered as additional design elements associated with learners’ engagement with AI feedback, while further research is needed to establish their effectiveness across different contexts. Overall, the results suggested that AI-generated feedback should be designed as an integrated cognitive–affective–metacognitive system in which different components complement one another.

### 4.10 Limitations and future research

This study has several limitations that should be considered when interpreting the findings. First, due to its cross-sectional design, all observed relationships should be interpreted as associative rather than causal. Accordingly, interpretations implying that AI feedback improves or reduces learner outcomes should be avoided given the study design, and future longitudinal or experimental studies are needed to examine temporal and causal relationships.

Although multiple mitigating steps were taken, common method variance could not be fully excluded due to the reliance on self-reported, single-source data regarding perceived engagement with ChatGPT-generated feedback without access to objective usage indicators. The study was conducted within a single institutional context in Saudi Arabia and focused on engineering students, limiting generalizability.

A further limitation concerns the dimensional structure of AI-generated feedback. Although discriminant validity was established using the Fornell–Larcker criterion and HTMT assessment, the results did not indicate high correlations among AIF, ESAF, and MSAF. Instead, the constructs demonstrated low empirical overlap, supporting their treatment as separate first-order constructs in the present model. Therefore, the decision not to test a higher-order model was based on the study objective of examining differentiated relationships among feedback dimensions rather than on evidence of strong construct similarity. Future research could compare competing measurement models, including higher-order, bifactor, and alternative specifications, to further clarify the structure of AI-generated feedback perceptions.

The proposed model could be extended by incorporating additional mediating and moderating factors, such as academic resilience, self-regulated learning, metacognitive awareness, AI literacy, digital competence, and learning mindset. Ethical, pedagogical, and governance dimensions of AI-supported feedback systems warrant further investigation as well. Finally, future studies could examine heterogeneity in usage patterns by differentiating between intensity, consistency, and quality of interaction with AI-generated feedback systems.

## Data Availability

The raw data supporting the conclusions of this article will be made available by the authors, without undue reservation.

## References

[ref1] Alsaiari O. Baghaei N. Lahza H. Lodge J. M. Boden M. Khosravi H. (2025). Emotionally enriched AI-generated feedback: supporting student well-being without compromising learning. Comput. Educ. 239:105363. doi: 10.1016/j.compedu.2025.105363

[ref2] Alshammari S. H. Alkhwaldi A. F. Alshammari A. E. A. Alshammari M. H. Alrashidi M. E. (2026). Extending the UTAUT model: the role of cognitive flexibility in AI adoption in higher education. Acta Psychol. 263:106363. doi: 10.1016/j.actpsy.2026.106363, 41633124

[ref3] Ba S. Yang L. Yan Z. Looi C. K. Gašević D. (2025). Unraveling the mechanisms and effectiveness of AI-assisted feedback in education: a systematic literature review. Comput. Educ. Open. 9:100284. doi: 10.1016/j.caeo.2025.100284

[ref4] Bautina M. (2025). Artificial intelligence in education: balancing personalized learning with cognitive and psychological risks. Pedag. Acad. Sci. Pap. 20:11896. doi: 10.5281/zenodo.16411896

[ref5] Begum R. Solangi M. W. Shaikh A. Ali A. (2025). The role of cognitive flexibility in adapting to climate change: AI-driven educational strategies for developing adaptive thinking. Crit. Rev. Soc. Sci. Stud. 3, 2500–2515. doi: 10.59075/he5g9j06

[ref6] Bentler P. M. Bonett D. G. (1980). Significance tests and goodness of fit in the analysis of covariance structures. Psychol. Bull. 88, 588–606. doi: 10.1037/0033-2909.88.3.588

[ref7] Chauncey S. A. McKenna H. P. (2024a). “Exploring the Potential of Cognitive Flexibility and Elaboration in Support of Curiosity, Interest, and Engagement in Designing AI-rich Learning Spaces, Extensible to Urban Environments,” in Distributed, Ambient and Pervasive. Interactions: 12th International Conference, DAPI 2024, Held as Part of the 26th HCI International Conference, HCII 2024, Washington, DC, USA, June 29–July 4, 2024, Proceedings, eds. Streitz N. A. Konomi S. (Cham, Switzerland: Springer Nature), 209–230.

[ref8] Chauncey S. A. McKenna H. P. (2024b). Creativity and innovation in civic spaces supported by cognitive flexibility when learning with AI chatbots in smart cities. Urban Sci. 8:16. doi: 10.3390/urbansci8010016

[ref9] Chen X. Xiao L. (2025). Serendipitous sparks: AI information encounter, cognitive flexibility, AI literacy, and university student creativity. Front. Psychol. 16:1623730. doi: 10.3389/fpsyg.2025.1623730, 41383430 PMC12689981

[ref10] Creswell J. W. Creswell J. D. (2018). Research Design: Qualitative, Quantitative, and Mixed Methods Approaches. 5th Edn Thousand Oaks, CA: Sage Publications.

[ref11] Deng K. Chen X. (2025). Exploring the impact of AI-driven emotional resilience on academic persistence, motivation, cognitive flexibility, and autonomy in self-regulated learning: a self-determination theory perspective. Learn. Motiv. 92:102167. doi: 10.1016/j.lmot.2025.102167

[ref12] Dennis J. P. Vander Wal J. S. (2010). The cognitive flexibility inventory: instrument development and estimates of reliability and validity. Cogn. Ther. Res. 34, 241–253. doi: 10.1007/s10608-009-9276-4

[ref13] Edmondson A. C. (1999). Psychological safety and learning behavior in work teams. Adm. Sci. Q. 44, 350–383. doi: 10.2307/2666999

[ref14] Elshanhaby W. Ahmed N. Noureldin A. Leila M. Abdelmutalib I. Aboueldahab M. . (2026). Unfolding the relationship between psychological safety, knowledge sharing, and innovation commitment in private higher education institutions in Egypt. Adm. Sci. 16:64. doi: 10.3390/admsci16020064

[ref15] Fan X. Yang N. (2026). When digital meets human: how organizational digital capability promotes service innovation via cognitive flexibility and empowering leadership. Acta Psychol. 262:106014. doi: 10.1016/j.actpsy.2025.106014, 41349271

[ref16] Field A. (2009). Discovering Statistics Using SPSS. 3rd Edn London: SAGE Publications Ltd.

[ref17] Fornell C. Larcker D. F. (1981). Evaluating structural equation models with unobservable variables and measurement error. J. Mark. Res. 18, 39–50. doi: 10.1177/002224378101800104

[ref18] Gross J. J. (1998). The emerging field of emotion regulation: an integrative review. Rev. Gen. Psychol. 2, 271–299. doi: 10.1037/1089-2680.2.3.271

[ref19] Hacıalioğlu N. Boyraz Şeker E. Kaya F. (2025). Nurses’ attitudes towards artificial intelligence: relationship between cognitive flexibility and emotion regulation. BMC Psychol. 13:1121. doi: 10.1186/s40359-025-03467-5, 41068976 PMC12513020

[ref20] Hair J. F. Anderson R. E. Tatham R. L. Black W. C. (2010). Multivariate Data Analysis. 5th Edn New Jersey, Englewood Cliffs: Prentice-Hall.

[ref21] Hair J. F. Hult G. T. M. Ringle C. M. Sarstedt M. (2022). A Primer on Partial Least Squares Structural Equation Modeling (PLS-SEM). 3rd Edn Thousand Oaks, CA: Sage Publications.

[ref23] Henseler J. Dijkstra T. K. Sarstedt M. Ringle C. M. Diamantopoulos A. Straub D. W. . (2014). Common beliefs and reality about PLS: comments on Rönkkö and Evermann (2013). Organ. Res. Methods 17, 182–209. doi: 10.1177/1094428114526928

[ref24] Henseler J. Hubona G. Ray P. A. (2016). Using PLS path modeling in new technology research: updated guidelines. Ind. Manag. Data Syst. 116, 2–20. doi: 10.1108/IMDS-09-2015-0382

[ref25] Henseler J. Ringle C. M. Sarstedt M. (2015). A new criterion for assessing discriminant validity in variance-based structural equation modeling. J. Acad. Mark. Sci. 43, 115–135. doi: 10.1007/s11747-014-0403-8

[ref27] Hohl K. Dolcos S. (2024). Measuring cognitive flexibility: a brief review of neuropsychological, self-report, and neuroscientific approaches. Front. Hum. Neurosci. 18:1331960. doi: 10.3389/fnhum.2024.1331960, 38439938 PMC10910035

[ref28] Hu L. T. Bentler P. (1999). Cutoff criteria for fit indexes in covariance structure analysis: conventional criteria versus new alternatives. Struct. Equ. Model. 6, 1–55. doi: 10.1080/10705519909540118

[ref29] Karaoglan Yilmaz F. G. K. Yilmaz R. Ustun A. B. Uzun H. (2025). Exploring the role of cognitive flexibility, digital competencies, and self-regulation skills on students’ generative artificial intelligence anxiety. Comput. Hum. Behav. Artif. Hum. 5:100187. doi: 10.1016/j.chbah.2025.100187

[ref30] Kiye S. Çiçek Habeş E. Ç. (2024). The mediating role of cognitive emotion regulation in the relationship between cognitive flexibility and psychological well-being. İnönü Üniv. Eğitim Fakült. Derg. 25, 1292–1310. doi: 10.17679/inuefd.1481952

[ref31] Kluger A. N. DeNisi A. (1996). The effects of feedback interventions on performance: a historical review, a meta-analysis, and a preliminary feedback intervention theory. Psychol. Bull. 119, 254–284. doi: 10.1037/0033-2909.119.2.254

[ref32] Kraft D. Rademacher L. Eckart C. Fiebach C. J. (2020). Cognitive, affective, and feedback-based flexibility—disentangling shared and different aspects of three facets of psychological flexibility. J. Cogn. 3:21. doi: 10.5334/joc.120, 32984758 PMC7500224

[ref33] McClintock A. H. Fainstad T. Blau K. Jauregui J. (2023). Psychological safety in medical education: a scoping review and synthesis of the literature. Med. Teach. 45, 1290–1299. doi: 10.1080/0142159X.2023.2216863, 37266963

[ref34] McGuire M. (2025). Feedback, reflection and psychological safety: rethinking assessment for student well-being in higher education. Assess. Eval. Higher Educ. 1, 1–25. doi: 10.1080/02602938.2025.2548590

[ref35] Medina Merodio J. A. Morales Chan M. Barchino Plata R. Amado-Salvatierra H. R. Hernandez-Rizzardini R. (2026). Impact of generative artificial intelligence feedback on online student satisfaction. Front. Comput. Sci. 7:1708114. doi: 10.3389/fcomp.2025.1708114

[ref36] Nagata N. Uetake T. (2025). “Enhancing Psychological Safety in Online Learning Environment with Generative AI,” in Blended Learning. Sustainable and Flexible Smart Learning: 18th International Conference on Blended Learning, ICBL 2025, Bangkok, Thailand, July 22–25, 2025, Proceedings, eds. Ma W. W. K. Cheung S. S. K. Li C. Prayadsab P. Mungwattana A. (Singapore: Springer Nature), 74–85.

[ref37] Na-Songkhla J. Mahakaew V. Peytcheva-Forsyth R. (2024). “The Emergence of Generative Artificial Intelligence: Enhancing Critical Thinking Skills in ChatGPT Integrated Cognitive Flexibility Approach,” in Generative Artificial Intelligence in Higher Education: A Handbook for Educational Leaders, ed. Na-Songkhla J. (Canada: JMIR Publications), 52–63.

[ref38] Pallant J. (2010). SPSS Survival Manual. 4th Edn New York: McGraw-Hill Companies.

[ref39] Peng Z. Wan Y. (2025). Human vs. AI: what makes students prefer to self-disclosure to AI teaching assistant? The effect of psychological safety and relationship norms. Educ. Inf. Technol. 30, 20851–20878. doi: 10.1007/s10639-025-13586-6

[ref40] Pop M. V. Tonț G. Flonta F. V. Flore M. (2025). Agentic AI in STEM education: enhancing cognitive flexibility and workforce readiness. BRAIN 16, 239–249. doi: 10.70594/brain/16.S1/20

[ref41] Shepard C. W. (2025). *Psychological Safety in Higher Education Faculty [Doctoral Dissertation]* University of Texas Tech.

[ref42] Tabachnick B. G. Fidell L. S. (2006). Using Multivariate Statistics. 5th Edn Boston, MA: Allyn and Bacon.

[ref43] Thompson B. Daniel L. G. (1996). Factor analytic evidence for the construct validity of scores: a historical overview and some guidelines. Educ. Psychol. Meas. 56, 197–208. doi: 10.1177/0013164496056002001

[ref44] Worapongpat N. (2025). The mindfulness in the use of artificial intelligence (AI) and its impact on learning for graduate students at private universities in Thailand. J. Intellect. Educ. 4, 80–92. Available online at: https://so06.tci-thaijo.org/index.php/IEJ/article/view/278862

[ref45] Xiong M. Chan N. N. Wong B. E. Xie X. Na M. (2025). Investigating cognitive flexibility and innovation in interdisciplinary project-based learning: the role of openness to learning and peer feedback quality in vocational education. Front. Psychol. 16:1691422. doi: 10.3389/fpsyg.2025.1691422, 41356025 PMC12676700

[ref46] Zeidan F. Johnson S. K. Diamond B. J. David Z. Goolkasian P. (2010). Mindfulness meditation improves cognition: evidence of brief mental training. Conscious. Cogn. 19, 597–605. doi: 10.1016/j.concog.2010.03.014, 20363650

[ref47] Zhao C. Q. Cao J. Lin J. Koedinger K. R. (2026). LLM-based multimodal feedback produces equivalent learning and better student perceptions than educator feedback. arXiv 2026:15280. doi: 10.48550/arXiv.2601.15280

