# Supplementary material for: Adaptive AI-generated feedback in higher education: exploring associations with cognitive flexibility and academic psychological safety among engineering students
Source: Front Psychol. 2026 Aug 31;17:1820714. doi: 10.3389/fpsyg.2026.1820714 (PMC13572651; doi:10.3389/fpsyg.2026.1820714)
Supplement: Supplementary file 1 [file Table_1.DOCX]

**Appendix 1: Measurement Instrument**

**Part one: Control Variables**

Gender, academic year, engineering discipline, prior experience with AI tools, and frequency of AI feedback use were included as control variables in the structural model.

**Part Two: Measurement Scales of Study Constructs**

| **Construct** | **Code** | **Survey Items** | **References** |
| --- | --- | --- | --- |
| **Section A: Adaptive AI-Generated Feedback (AIF):** | AIF1 | The AI-generated feedback I receive is tailored to my learning needs. | Ba et al. (2025); Chauncey and McKenna (2024a); Nagata and Uetake (2025). |
|  | AIF2 | The feedback helps me understand how to improve, not just what is wrong. | Ba et al. (2025); Nagata and Uetake (2025). |
|  | AIF3 | The AI feedback encourages me to think about alternative solutions or approaches. | Ba et al. (2025). |
|  | AIF4 | The feedback is delivered in a non-judgmental and supportive manner. | Chauncey and McKenna (2024a). |
|  | AIF5 | I feel comfortable revising my work multiple times based on AI feedback. | Ba et al. (2025); Nagata and Uetake (2025). |
|  | AIF6 | The AI feedback helps me reflect on my problem-solving process. | Ba et al. (2025). |
|  | AIF7 | The feedback supports my learning without making me feel evaluated or criticized. | Chauncey and McKenna (2024a). |
| **Section B: Cognitive Flexibility (CF)** | CF1 | I can easily change my problem-solving approach when my first solution does not work. | Kraft et al. (2020); Pop et al. (2025). |
|  | CF2 | I consider multiple perspectives when solving engineering problems. | Hohl & Dolcos (2024). |
|  | CF3 | I am open to revising my ideas after receiving feedback. | Kraft et al. (2020). |
|  | CF4 | I can adapt my thinking when learning tasks become more complex. | Pop et al. (2025). |
|  | CF5 | I am comfortable exploring alternative solutions suggested by AI feedback. | Ba et al. (2025); Pop et al. (2025). |
|  | CF6 | I can connect new information with what I already know in different ways. | Hohl & Dolcos (2024). |
|  | CF7 | I view mistakes as opportunities to rethink my approach. | Kraft et al. (2020). |
| **Section C: Academic Psychological Safety (APS)** | APS1 | I feel safe asking questions when using AI-supported learning tools. | McClintock et al. (2023). |
|  | APS2 | I am not afraid of making mistakes when interacting with AI-generated feedback. | McGuire (2025). |
|  | APS3 | I feel comfortable expressing confusion or uncertainty to the AI system. | McClintock et al. (2023). |
|  | APS4 | Using AI feedback reduces my anxiety about being evaluated. | McGuire (2025); Peng & Wan (2025). |
|  | APS5 | I feel encouraged to experiment with different ideas during learning activities. | Edmondson (1999). |
|  | APS6 | I can openly explore new approaches without fear of failure. | Peng & Wan (2025). |
|  | APS7 | AI feedback makes learning feel less stressful and more supportive. | McGuire (2025); Peng & Wan (2025). |
| **Section D: Emotionally Supportive AI Feedback (ESAF)** | ESF1 | The AI feedback helps me stay calm when I struggle with a task. | Deng et al. (2025); Gross (1998). |
|  | ESF2 | The feedback encourages me to view challenges in a positive way. | Gross (1998); Nagata & Uetake (2025). |
|  | ESF3 | The AI responds in a tone that feels empathetic and understanding. | Chauncey and McKenna (2024a); Deng et al. (2025). |
|  | ESF4 | The feedback helps me reframe mistakes as part of the learning process. | Gross (1998). |
|  | ESF5 | I feel emotionally supported when receiving AI-generated feedback. | Deng et al. (2025); Nagata & Uetake (2025). |
| **Section E: Mindfulness-Supportive AI Feedback (MSAF)** | MSF1 | The AI feedback encourages me to pause and reflect before responding. | Kiye & Çiçek Habeş (2024); Zeidan et al. (2010). |
|  | MSF2 | The feedback helps me stay focused on the current learning task. | Kiye & Çiçek Habeş (2024); Worapongpat (2025). |
|  | MSF3 | I become more aware of my thinking process when using AI feedback. | Worapongpat (2025); Zeidan et al. (2010). |
|  | MSF4 | The feedback supports self-regulation rather than rushing to answers. | Kiye & Çiçek Habeş (2024); Zeidan et al. (2010). |
|  | MSF5 | AI feedback helps me stay engaged without feeling overwhelmed. | Kiye & Çiçek Habeş (2024); Worapongpat (2025). |

**Note.** All measurement items were adapted from previously validated scales (see references column) with minor wording modifications to fit the AI-based learning context.

**References:**

Ba, S., Yang, L., Yan, Z., Looi, C.K., and Gašević, D. (2025). Unraveling the mechanisms and effectiveness of AI-assisted feedback in education: A systematic literature review. Comput. Educ. Open. 9, 100284. doi: [10.1016/j.caeo.2025.100284](https://doi.org/10.1016/j.caeo.2025.100284).

Chauncey, S.A., and McKenna, H.P. (2024b). Creativity and innovation in civic spaces supported by cognitive flexibility when learning with AI chatbots in smart cities. Urban Sci. 8, 16. doi: [10.3390/urbansci8010016](https://doi.org/10.3390/urbansci8010016).

Deng, K., and Chen, X. (2025). Exploring the impact of AI-driven emotional resilience on academic persistence, motivation, cognitive flexibility, and autonomy in self-regulated learning: A self-determination theory perspective. Learn. Motiv. 92, 102167. doi: [10.1016/j.lmot.2025.102167](https://doi.org/10.1016/j.lmot.2025.102167).

Gross, J.J. (1998). The emerging field of emotion regulation: An integrative review. Rev. Gen. Psychol. 2, 271-299. doi: [10.1037/1089-2680.2.3.271](https://doi.org/10.1037/1089-2680.2.3.271).

Hohl, K., and Dolcos, S. (2024). Measuring cognitive flexibility: A brief review of neuropsychological, self-report, and neuroscientific approaches. Front. Hum. Neurosci., 18, 1331960.‏ doi: [10.3389/fnhum.2024.1331960](https://doi.org/10.3389/fnhum.2024.1331960)

Kiye, S., and Çiçek Habeş, E.Ç. (2024). The mediating role of cognitive emotion regulation in the relationship between cognitive flexibility and psychological well-being. İnönü Üniv. Eğitim Fak. Derg. 25, 1292-1310. doi: [10.17679/inuefd.1481952](https://doi.org/10.17679/inuefd.1481952).

Kraft, D., Rademacher, L., Eckart, C., and Fiebach, C.J. (2020). Cognitive, affective, and feedback-based flexibility—Disentangling shared and different aspects of three facets of psychological flexibility. J. Cogn. 3, 21. doi: [10.5334/joc.120](https://doi.org/10.5334/joc.120).

McClintock, A.H., Fainstad, T., Blau, K., and Jauregui, J. (2023). Psychological safety in medical education: A scoping review and synthesis of the literature. Med. Teach. 45, 1290-1299. doi: [10.1080/0142159X.2023.2216863](https://doi.org/10.1080/0142159x.2023.2216863).

McGuire, M. (2025). Feedback, reflection and psychological safety: Rethinking assessment for student well-being in higher education. Assess. Eval. Higher Educ., 1-25. doi: [10.1080/02602938.2025.2548590](https://doi.org/10.1080/02602938.2025.2548590).

Nagata, N., and Uetake, T. (2025). Enhancing psychological safety in online learning environment with generative AI, in Blended Learning. Sustainable and Flexible Smart Learning: 18th International Conference on Blended Learning, ICBL 2025, Bangkok, Thailand, July 22-25, 2025, Proceedings, eds. W. W. K. Ma, S. S. K. Cheung, C. Li, P. Prayadsab, and A. Mungwattana (Singapore: Springer Nature), 74-85. doi: [10.1007/978-981-96-8430-4_6](https://doi.org/10.1007/978-981-96-8430-4_6).

Peng, Z., and Wan, Y. (2025). Human vs. AI: What makes students prefer to self-disclosure to AI teaching assistant? The effect of psychological safety and relationship norms. Educ. Inf. Technol. 30, 20851-20878. doi: [10.1007/s10639-025-13586-6](https://doi.org/10.1007/s10639-025-13586-6).

Pop, M.V., Tonț, G., Flonta, F.V., and Flore, M. (2025). Agentic AI in STEM education: Enhancing cognitive flexibility and workforce readiness. BRAIN: Broad Res. Artif. Intell. Neurosci. 16(Sup. 1), 239-249. doi: [10.70594/brain/16.S1/20](https://doi.org/10.70594/brain/16.s1/20).

Worapongpat, N. (2025). The mindfulness in the use of artificial intelligence (AI) and its impact on learning for graduate students at private universities in Thailand. J. Intellect Educ. 4, 80-92. <https://so06.tci-thaijo.org/index.php/IEJ/article/view/278862>.

Zeidan, F., Johnson, S.K., Diamond, B.J., David, Z., and Goolkasian, P. (2010). Mindfulness meditation improves cognition: Evidence of brief mental training. Conscious. Cogn. 19, 597-605. doi: [10.1016/j.concog.2010.03.014](https://doi.org/10.1016/j.concog.2010.03.014).
